# The diversity of smallholder chicken farming in the Southern Highlands of Tanzania reveals a range of underlying production constraints

**DOI:** 10.1016/j.psj.2022.102062

**Published:** 2022-07-26

**Authors:** Wilson C. Wilson, Maja Slingerland, Simon Oosting, Frederick P. Baijukya, Anne-Jo Smits, Ken E. Giller

**Affiliations:** ⁎ Plant Production Systems Group, Wageningen University, 6700 AK Wageningen, the Netherlands; † Animal Production Systems Group, Wageningen University, 6700 AH Wageningen, the Netherlands; ‡ International Institute of Tropical Agriculture (IITA), P.O. Box 34441, Dar es Salaam, Tanzania; § Tanzania Livestock Research Institute (TALIRI), Uyole Centre, P.O. Box 6191, Mbeya, Tanzania; # Poultry Expertise Centre, Aeres, 3771 RN Barneveld, the Netherlands

**Keywords:** farm diversity, chicken feed, intensification, poultry management, problem tree

## Abstract

The poultry industry in Tanzania has grown steadily over the past decade. We surveyed 121 chicken farming households along an intensification gradient from backyard to semi-intensive and intensive production systems based on rearing system and assumed purpose and poultry breed in the Iringa region. About 30% of households had more than one breed and/or rearing system combination. The subdivision of poultry systems was refined by adding the size of the flocks to highlight variation in scale of operations. On this basis we distinguished 3 main types: 1) subsistence small-scale free-range chicken production; 2) market-oriented small to medium scale semi-intensive and 3) small to medium-large scale intensive systems. ‘Intensification’ involves the transition from keeping indigenous chickens to improved dual-purpose and exotic breeds driven by greater productivity and potential for income generation. The more intensive the production system, the more the intensity and diversity of diseases identified by farmers as their main problem, which was partly attributed to the greater sensitivity of the improved breeds, poor veterinary measures, and the high chicken density facilitating disease spread. Based on the survey we constructed a problem tree to classify the underlying constraints and their interrelations, and to identify common root causes, based on which we propose practical solutions to improve chicken production. Development of medium-large scale systems is particularly constrained by a limited supply of 1-day-old chicks and theft. By contrast, intensification of small-scale systems is constrained by limited access to quality feed, vaccines and medicines, capital, and lack of a reliable market, partly due to the absence of farmer organization. These constraints can be addressed through formation of producer groups and promotion of outgrower and enterprise development models. Enterprise development appears to be the most promising business model for smallholder chicken farmers given that it allows farmers more freedom in decision-making and management while strengthening linkages with input suppliers and output markets to ensure a viable and profitable business.

## BACKGROUND

Driven by rapid urbanization (Africapolis, 2019), economic growth, and change in consumer's affluence, the demand for animal-sourced food (**ASF**) in Tanzania, particularly chicken meat is projected to increase by 148%, while that of beef, goat and mutton, pork and milk will increase by 87, 71, 88, and 108%, respectively from the mid-2000s to 2030 (Baker et al., 2016). Currently, the demand for chicken meat and eggs in Tanzania already strongly exceeds domestic production and supply, mainly due to low production and productivity of indigenous chicken breeds and limited availability of quality feed (Naggujja et al., 2020). Policy interventions taken to improve the poultry sector in Tanzania include the genetic improvement of the indigenous chickens raised under a low-input free-range system (Michael et al., 2018). As a result, improved dual-purpose chickens have been introduced in various regions of the country over the past decade (Sanka et al., 2020). In the same period, the number of commercial layers and broilers raised under high input-output intensive systems has been increasing, particularly in urban areas (Sindiyo et al., 2018; Mushi et al., 2020).

Chickens are important sources of animal-sourced food in Tanzania for numerous reasons. First, 86% of the livestock keeping households in the country own chickens (MLDF, 2015). About 80% of the chickens are owned by women who have control over decisions on sales and consumption of chicken meat and eggs (Galiè et al., 2015; Tavenner et al., 2019; Shapa et al., 2021). Second, chickens are sold alive and do not necessarily need central slaughterhouses and cold chains. Third, a chicken is a unit fit for rural household consumption compared with ruminants which generate too much meat to be consumed in one meal. Chicken eggs are also appropriate units for daily consumption and can be stored for some days without cooling. Fourthly, managing a chicken enterprise is relatively easy, requiring a small capital investment with promising income generation within a short period of time and hence, attracting more women and youth (Hundie et al., 2019; Ngongolo and Chota, 2021). White meat including that of chicken is considered a healthier food than red meat, and therefore, the trend of consumption is expected to increase steadily (Weber and Windisch, 2017). In addition, chickens play important roles in satisfying religious and social cultural needs in most rural communities in the country (Akinola and Essien, 2011).

From a regional perspective, the Southern Highlands of Tanzania have great potential in food production and are considered to be the country's breadbasket (Altare et al., 2016). Despite the potential of the Southern Highlands in food production, there are high rates of stunting among under-five children, mainly associated with limited dietary diversity among households (Ministry of Health et al., 2016). Previous research on chicken production in the Southern Highlands focused on the characterization of the indigenous chicken breeds (Guni and Katule, 2013; Guni et al., 2013; Mwambene et al., 2019); and, more recent on the performance evaluation of the newly introduced crossbred namely Sasso and Kuroiler, implemented by the African Chicken Genetic Gain project (Pius and Mbaga, 2018; Andrew et al., 2019). These aforementioned studies focused on chicken production raised under rural outset, with scant information on the intensification gradient, dynamics, and constraints in different production systems.

We set out to understand chicken farming diversity and explore the intensification gradient from backyard, semi-intensive and intensive production systems and the underlying constraints limiting production in urban and rural areas. The findings suggest relevant innovation options to improve the domestic production of meat and eggs in the identified systems that will contribute to improving access to animal-sourced food to support diverse diets among households in Tanzania.

## METHODOLOGY

### Description of the Study Area

To understand the current chicken production systems and challenges faced by farmers, we conducted a household survey in the Iringa region, SH of Tanzania from November to December 2018. For the representation of urban and rural locations, three administrative districts were selected from the Iringa region namely Iringa municipality with urban farmers and Kilolo and Iringa districts with rural chicken farmers (Figure 2). The districts were selected based on the intensification gradient we observed earlier in urban and rural areas, potential in the commercialization of chicken production, the existence of grain millers, feed processors, and hatcheries established over the past decade (Wilson et al., 2021). The three districts comprised 60 administrative wards in urban and rural, respectively. Based on the government designations on urbanization, population density and economic advancement the wards located in Iringa Municipality were considered as the representatives of the urban location when compared to Iringa rural and Kilolo districts (Africapolis, 2019; Wineman et al., 2020).

**Figure 1:**
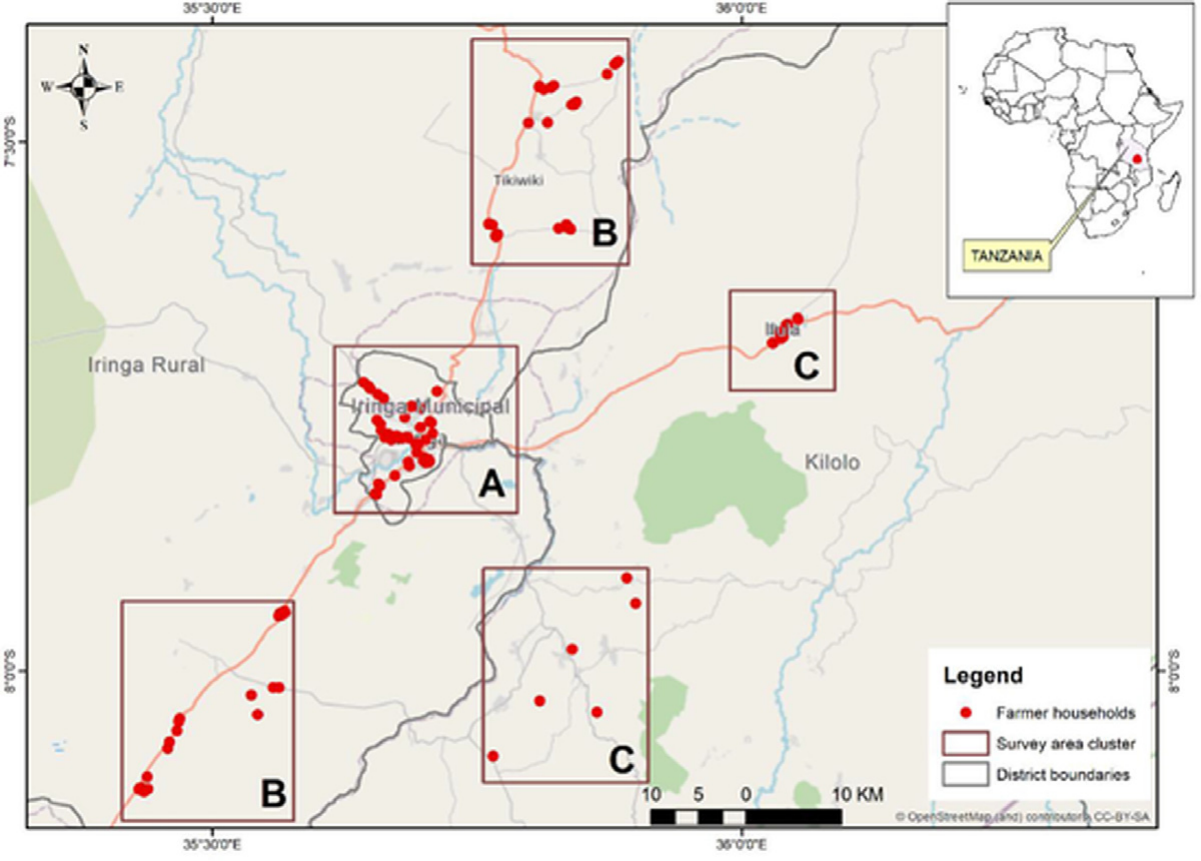
Location of 121 chicken farming households interviewed in Iringa Municipality (A) Iringa rural (B) and Kilolo districts in the Iringa region.

### Sampling Framework and Data Collection

In total, 121 poultry farmers were interviewed in urban and rural locations (Figure 1) using semistructured questionnaires that were programmed using Open Data Kit (**ODK**). Seventeen out of 60 wards were purposively selected from the three districts based on having a relatively high number of chickens according to the district statistics and on discussions with the respective district extension offices. Since the exact number of farmers keeping chicken in the respective wards was not available, the farmers were randomly sampled from the list provided by the respective ward extension officers and interviews conducted depending on their availability and willingness to participate. In total, 48 and 73 chicken farming households were interviewed to explore the diversity in the farming systems in urban and rural locations, respectively. The households interviewed were keeping indigenous, improved crossbred and/or exotic chickens. Preliminary backyard, semi-intensive and intensive systems were distinguished based on a gradient of increasing controlled environments in terms of housing, feeding, and veterinary care (see also Wilson et al., 2021) to check whether all types were represented in our sample.

To characterize the different farm types, a semistructured questionnaire was administered collecting information on 1) general characteristics of the household that is, demographics and socioeconomic activities, farm size; 2) chicken production and management practices; (i.e., housing, feeds and feeding, health, disease, and parasite control), chicken breeds and types, flock size, egg production per week, experience in keeping chicken, rearing systems, sources of chicken flock; 3) product handling, marketing, and consumption; and 4) challenges limiting chicken production. The interviews were conducted on the farms in Kiswahili by 6 enumerators.

### Data Analysis

The quantitative data resulting from the interviews were analyzed using Statistical Package for Social Sciences (**SPSS**) version 25 to obtain descriptive statistics (frequency counts, median, maximum, and minimum numbers) of the surveyed sample which were used to characterize the farm types (Table 1). The significance of the mean differences in number of chicken and productivity in urban and rural was assessed using Analysis of Variances (**ANOVA**) and Chi-square test (Table S1). The farming system was primarily grouped into backyard/free-range system, semi-intensive, and intensive systems based on the rearing systems that is, housing and feeding (Chaiban et al., 2020). Backyard is scavenging outdoor, semi-intensive is partly indoor and fed, intensive is permanent indoor and fed. The farm types were further subcategorized based on the breeds of chicken (indigenous, improved cross, and exotic), and purpose of keeping chicken (meat, eggs and dual-purpose [meat and eggs]). In the next step, the predefined farm types were further subdivided into small-scale, medium-scale, medium-large scale production based on the number of chickens as partly applied by Chaiban et al. (2020). We categorized small scale semi-intensive systems as having less than 50 chickens and only indigenous chickens and we labelled as intensive small scale types with less than 150 chickens. Semi-intensive and intensive systems with larger numbers of improved breeds up to 500 median are called medium scale. As individual intensive systems may have up to 5,700 chickens we call them medium to large scale system.

**Table 1:** Description of the households sampled and interviewed for the characterization of chicken farming in urban and rural, Iringa region.

Farm location		Urban (n = 48)							Rural (n = 73)					
Households interviewed per dominant rearing system*		Free-range (n = 11)	Semi-intensive (n = 5)			Intensive (n = 31)			Free-range (n = 29)	Semi-intensive (n = 25)		Intensive (n = 26)		
Chicken breed		Indigenous	Indigenous	Improved cross	Exotic	Indigenous	Improved cross	Exotic	Indigenous	Indigenous	Improved cross	Indigenous	Improved cross	Exotic
Number of systems found		11	4			17			29	24		17		
				1			18				3		12	
					1			12						5
Purpose of keeping chicken	Meat	1	2			5	5	5	1	3	1		5	3
	Eggs	1			1	7	11	11		1	1		4	3
	Meat and eggs	9	4	1		17	14		28	24	3	17	10	
Flock size per system^1^		19	31	96	422	145	183	500	23	30	170	50	276	340
		(7–86)	(7–422)			(16–1,208)	(35–1,208)	(150–5,700)	(2–215)	(7–294)	(157–294)	(18–498)	(86–850)	(209–850)
Number of chickens sold/month^1^		2	2			9	10	20	2	2	20	4	15	57
		(0–12)	(1–2)			(3–167)	(3–167)	(150–5,700)	(0–12)	(0–30)	(20–28)	(0–53)	(0–64)	(10–64)
Number of chickens retained for home consumption/month^1^		1	1	1	1	1	2	2	1	2	2	1	2	4
		(0–2)	(0–1)			(0–18)	(1–4)	(0–167)	(0–2)	(0–2)	(1–2)	(0–4)	(1–5)	(1–5)
Number of eggs production/week^1^		12	15		20	150	250	381	16	17	205	30	350	405
		(5–50)	(7–25)			(7–2,170)	(7–2,170)	(86–11,970)	(0–140)	(7–130)	(130–280)	(5–559)	(0–2,940)	(0-2,940)
Number of eggs sold/week^1^		5	5		2	95	240	185	5	10	175	10	310	280
		(0–45)	(2–20)			(0–735)	(0–1,500)	(0–11,940)	(0–60)	(0–196)	(100–250)	(0–350)	(0–2,900)	(0–2,900)
Number of eggs retained for home consumption/week^1^		4				10	14	15	4	4	23	5	20	19
		(0–6)	(0–5)			(4–30)	(0–30)	(0–30)	(0–10)	(0–30)	(15–30)	(0–30)	(0–30)	(15–30)

1 Median, minimum and maximum.

⁎ One household may have more than one rearing system and/or breed.

**Figure 2:**
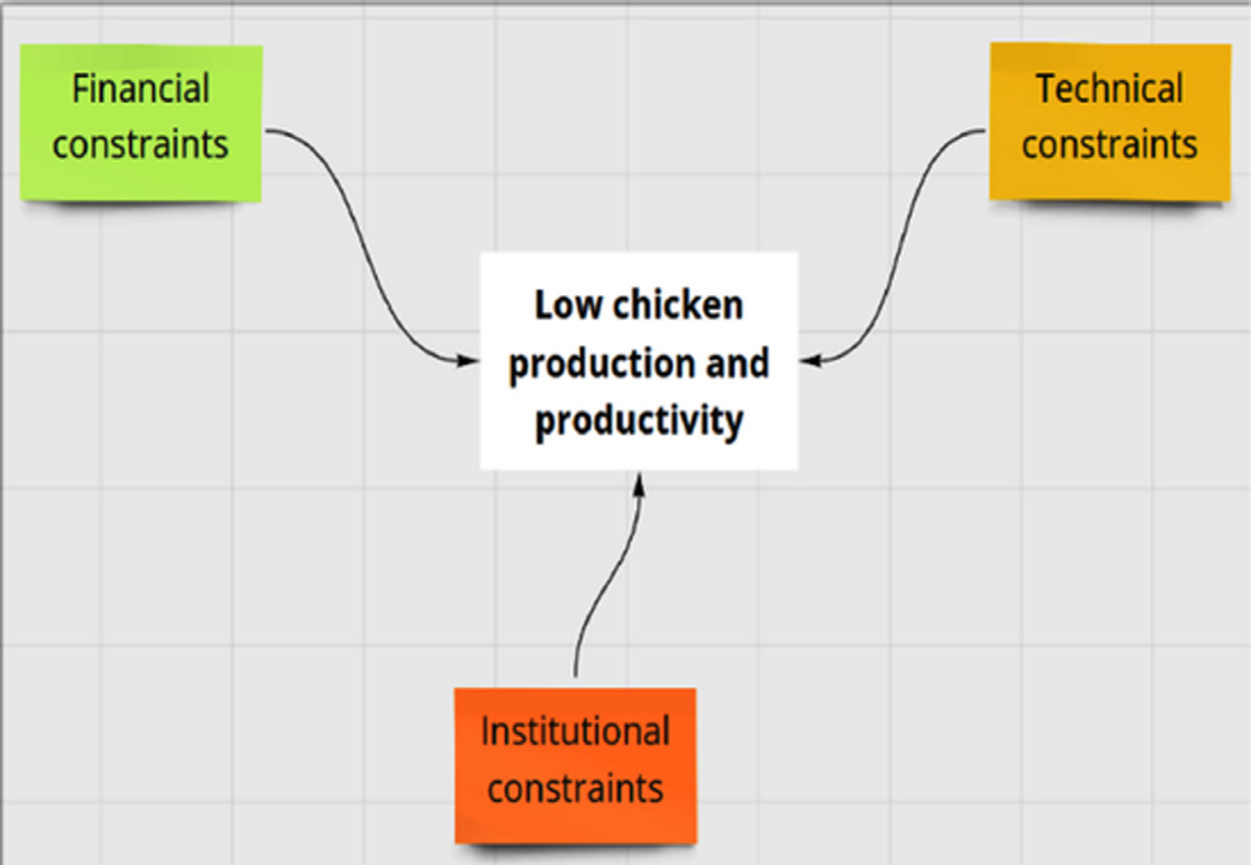
The conceptual framework for analyzing constraints in chicken production and productivity categorized into financial, technical, and institutional constraints indicated by green, yellow and orange boxes, respectively.

The constraints faced by chicken farmer based on the household interviews were coded and grouped into 3 major categories, that is, financial, technical, and institutional constraints using the analytical framework (Figure 2) of (Gale et al., 2013). The financial constraints are those related to the factors determining the total farm/firm size (Ringo and Lekule, 2020), while the technical constraints relate to farmers knowledge on chicken production and management that is, veterinary measures, feed and feeding practices (Mapiye et al., 2008; Justus et al., 2013; Mutua et al., 2019). Other technical aspects investigated included knowledge and awareness of farmers on record keeping, entrepreneurship and marketing skills and gender roles in different managerial aspects at the household level. The institutional aspects included the public support services and physical infrastructures (i.e., roads, water supply, communication technology, energy), market infrastructures, finance, and credit facilities (Mapiye et al., 2008).

In a next step, we conducted a problem tree analysis by classifying the constraints in the three earlier identified categories (recognizable by color) and assessing the relations between the identified constraints and their consequences. The problem tree distinguishes between the more immediate and root causes of low chicken production. Finally, the problem tree allowed finding practical solutions to increase chicken production at each of these levels and in each of the 3 earlier-mentioned categories.

## RESULTS

### Chicken Production System Types in Urban and Rural Locations

**Figure 3:**
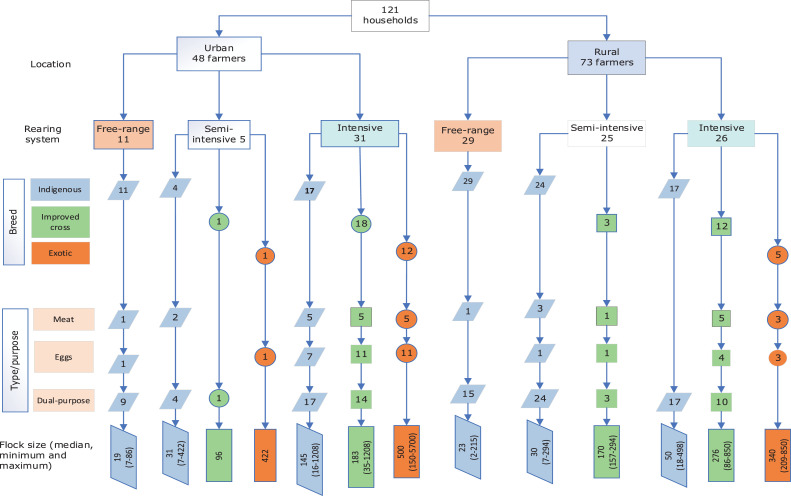
Subdivisions of the farm types identified in urban and rural, Iringa region. N.B: Some households raised multiple breeds of chicken/rearing systems

**Figure 4:**
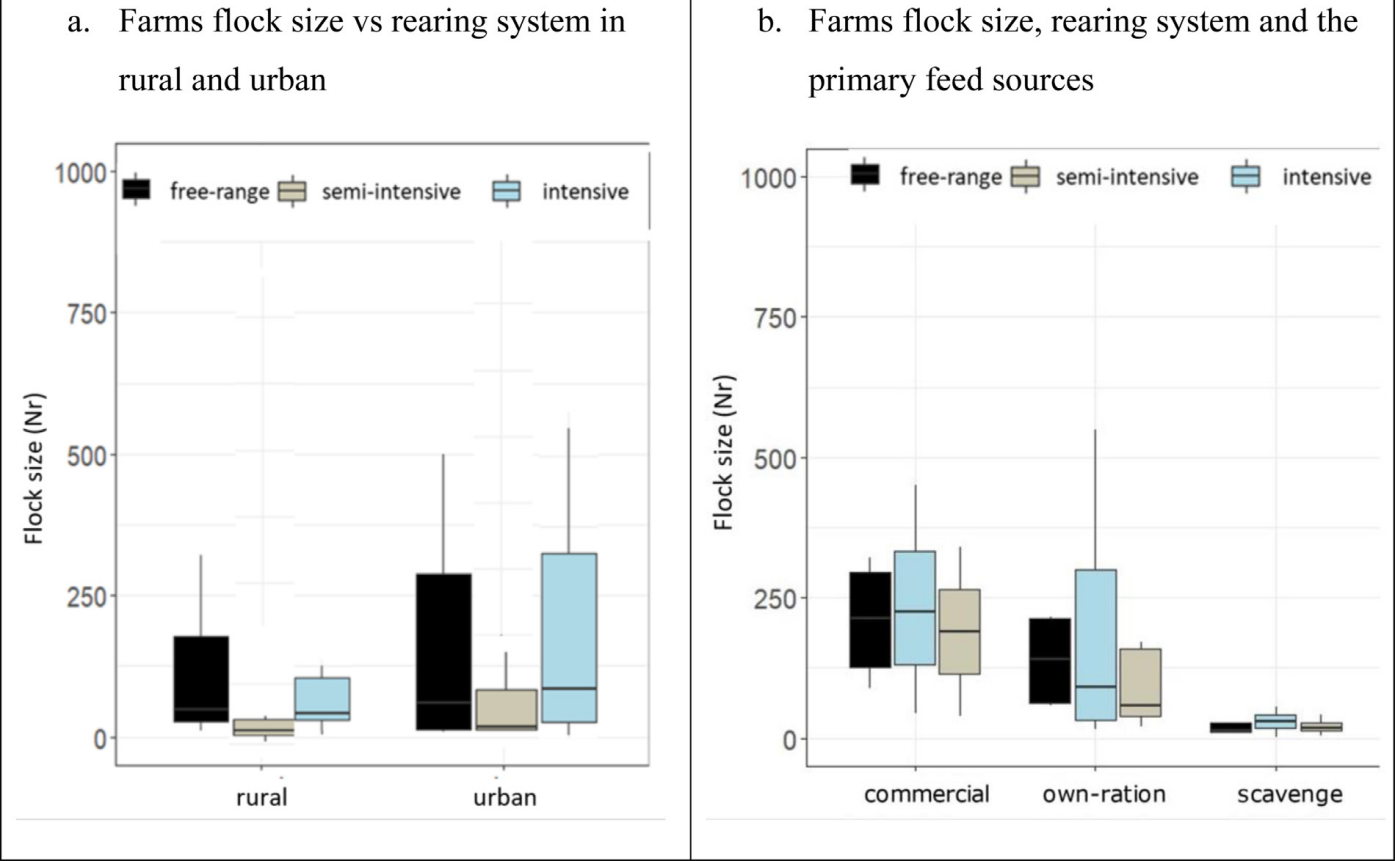
Boxplots of the quantitative (instant flock size [mean ± SE]) and qualitative variables farm location (A) and rearing system and primary feed sources (B).

We confirmed the existence of the 3 main production systems that had been initially proposed (Wilson et al., 2021); that is, free-range/extensive production, semi-intensive, and intensive systems both in urban and rural locations. Nevertheless, we felt the need to make subdivisions within these types based on the outcome of our survey. Characterization of the farming systems was primarily based on the rearing system (housing and feeding), breed of chicken, the purpose of keeping chickens and flock size (Figures 3 and 4). We found that 85% of the interviewed households raised indigenous chickens both for meat and eggs while fewer households raised the improved cross (28%) and exotic breeds (15%) for meat or eggs. Irrespective of the systems the flock size varied significantly between urban and rural locations *(P* < 0.05*)* with larger numbers of chickens produced and consumed in urban locations (Table S1). Indigenous chicken flocks consisted of chicks hatched at the farm and/or purchased from neighbours while the improved cross and exotic 1-day-old chicks were purchased from selling agents and hatcheries in the region.

### Type 1: Free Range: Small-Scale Systems (40)

The first type comprised smallholder farms raising indigenous backyard chicken in urban (23%) and rural (40%) locations. The farming households within this farm type had small flock sizes of about 19 and 23 chickens in urban and rural, respectively (Figure 3). In free-range farms, chickens rely on scavenging for their feed (Figure 4B). Chickens were raised for both eggs and meat, producing about 12 and 16 eggs per week in urban and rural, respectively. In an urban location, 42% of the produced eggs were sold and 33% were retained for home consumption. In rural locations, 31% of the produced eggs were sold and 22% were retained for home consumption. The remaining eggs were left for natural hatching. On average, the farming households sold about 2 chickens and consumed one chicken per month in both urban and rural.

### Type 2: Semi-intensive

#### Small-Scale Semi-intensive Systems (28)

This category constitutes farms raising, on average, 30 indigenous chickens, mainly in rural locations (86%) (Figure 3; Table 1). These chickens were partly scavenging outdoors and partly supplemented with own-made feed rations (Figure 4B). In this system, chickens were raised for both eggs and meat, producing about 17 eggs per week, of which 59% were sold and 24% retained for home consumption. Despite the chickens being supplemented with homemade feed, the production and productivity were the same as for farm type 1 above. On average, both urban and rural farming households sold and/or consumed about 2 chickens per month.

#### Medium-Scale Semi-intensive System (4)

This category constitutes the farms raising, on average, 170 improved cross-bred chickens, mainly in rural locations (75%). Chickens were partly raised outdoors and fed with commercial and/or homemade feed rations (Figure 4B). In this system, chickens were raised for both eggs and meat, producing about 205 eggs per week, of which 85% were sold and 11% retained for home consumption. On average, a medium-scale semi-intensive farming household sold about 20 chickens and consumed 2 chickens per month (Table 1).

### Type 3: Intensive Systems

#### Small-Scale Intensive Systems (34)

An equal number of small-scale intensive farming households were interviewed in both urban and rural locations. Farms keep indigenous dual-purpose chickens with a flock size of about 50 and 145 chickens in rural and urban locations, respectively (Table 1), raised for both meat and eggs. Chickens were raised and fed indoors whereby the small-scale intensive farms in rural locations fed their chickens with homemade feed rations while those in urban locations relied on a combination of homemade and commercial feed (Figure 4). Chickens were more productive in urban locations producing a large number of eggs per household per week (5 times higher compared to rural locations (Table 1). The number of eggs sold and consumed per week was also greater in urban households compared with rural ones.

#### Medium-Large Scale Intensive Systems (47)

This subcategory is comprised of farms keeping the improved cross-bred and exotic chickens in urban and rural locations. The mean flock sizes for the improved cross-bred were 183 and 275 in urban and rural, and 500 and 340 for the exotic chickens in urban and rural locations, respectively (Table 1). Chickens were raised indoors and fed with commercial and/or homemade feed (Figure 4B). About 71% of the interviewed farmers that were keeping exotic chickens were in an urban location with the majority raising exotic layers (65%). The farms keeping the improved cross-bred chickens produced about 250 and 350 eggs per week in urban and rural while the farms keeping the exotic layers produced about 381 and 405 eggs per week in urban and rural locations, respectively. The farms were run commercially where most eggs were sold and about 14 and 20 eggs were retained for household consumption per week in urban and rural locations, respectively. The households keeping the exotic chicken in rural areas retained 5 chickens for home consumption per month, compared with 2 chickens in all other farm types.

***Households** K**eeping** M**ultiple******B******reeds of Chickens in******O******ne or******M******ore******R******earing Systems (38)*** Out of the 121 interviewed households, 31% raised multiple breeds/types of chickens in one or more rearing systems in both urban and rural (Table S2). We found that the more intensified the system the more diverse the breeds of chickens were raised for both meat and eggs. The first subcategory, constitute 7 small-scale farms keeping indigenous dual-purpose chickens in multiple systems that is, free-range and semi-intensive system (4 farms), free-range and intensive system (3 farms). The second subcategory constitutes 4 medium-scale farms keeping improved crossbred chickens under medium scale and a small number of traditional dual-purpose chickens under the semi-intensive system, both for meat and eggs. The third subcategory constitutes medium-large scale intensive farms raising the improved crossbred and exotic chickens in combination with other breeds in multiple systems. Out of 30 households raising the improved dual-purpose chickens under the intensive system, 20% raised a large number of exotic chickens and 97% raised a small number of indigenous dual-purpose chickens under the free-range system (48%), semi-intensive (10%), and intensive systems (33%) both for meat and eggs. Out of 17 households raising exotic chickens under the intensive system, 53% raised indigenous and/or improved dual-purpose chickens both for meat and eggs.

### Constraints Limiting Chicken Production

**Figure 5:**
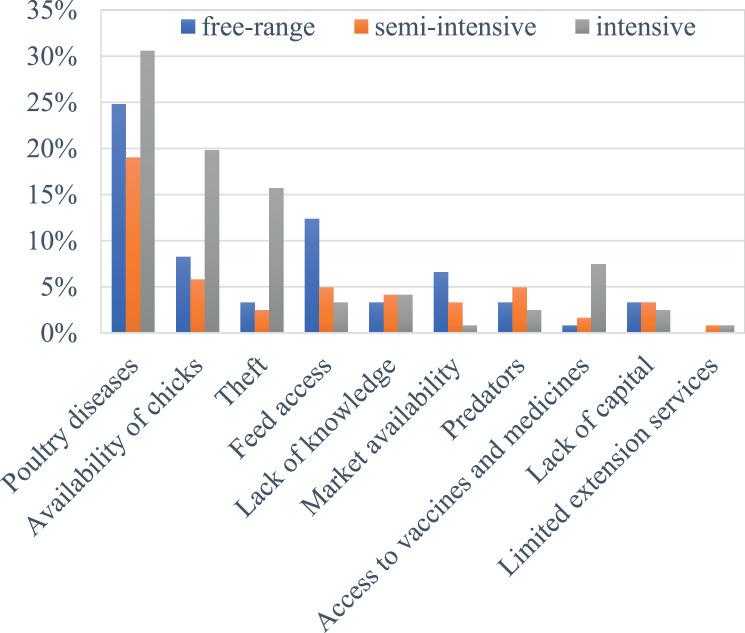
Constraints limiting chicken production in the Iringa region based on the household interviews.

The major constraints limiting chicken production identified during the household interviews include chicken diseases, poor availability of day-old chicks, theft and limited access to quality feed and/or feed ingredients (Figure 5). Other challenges include limited knowledge on managerial practices, market availability, predators, limited access to vaccines and medicines, lack of capital, and limited extension services. Some of the constraints varied among the production systems as further explained below.

#### Health Management

**Figure 6:**
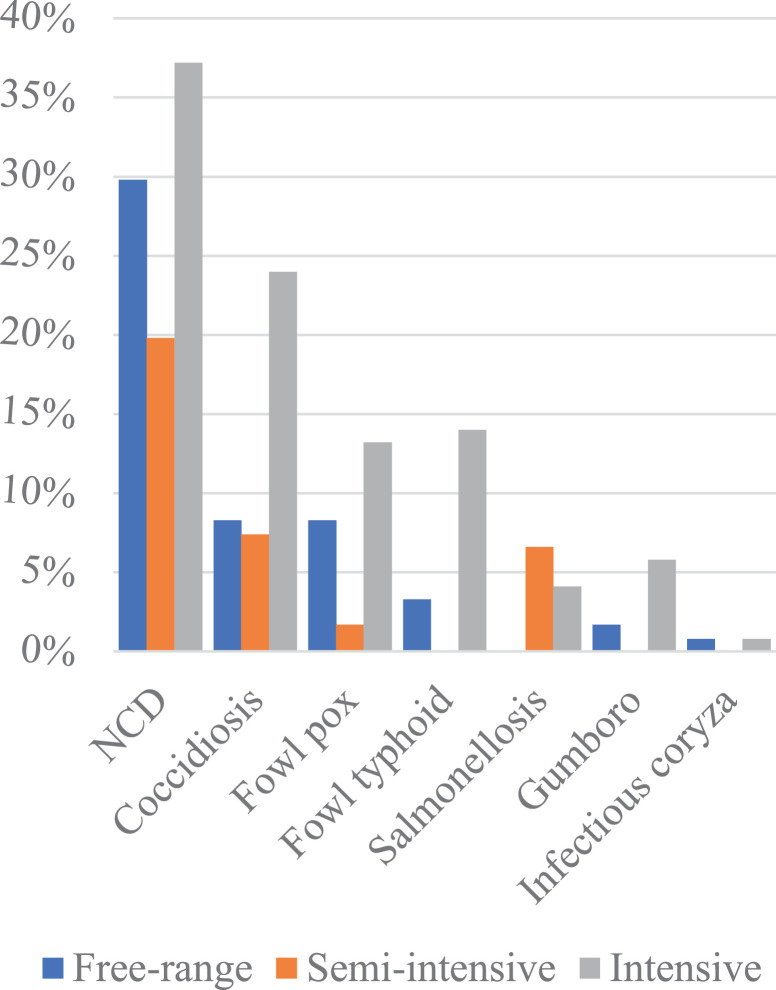
Occurrence of chicken diseases in the different production systems. Abbreviations: NCD, Newcastle disease.

We found that the more intensive the system, the more farmers highlighted disease problems, and also more types of diseases as their main problem (Figures 5 and 6). Newcastle disease (**NCD**) was the most prominent disease reported in the intensive (37%), free-range (30%) and semi-intensive systems (20%) followed by coccidiosis (13%), fowl pox (8%), and fowl typhoid (6%), each mostly reported in the intensive system. Other diseases included salmonellosis (4%), Gumboro (3%), and infectious coryza (2%).

#### Access to Chicken Feeds and Feed Ingredients

Limited access to quality feed was among the technical constraints limiting the free-range scavenging system partly related to the farmers’ lack of knowledge on how to formulate their own feed rations, limited access to feed ingredients (less available or too expensive) and lack of capital to buy the 50 kg packages of commercial feed. The primary sources of feed were strongly determined by flock size and the rearing system, whereby large flocks (>50 chickens) were fed on either commercial or locally-made feed rations under free-range, semi-intensive, and intensive systems. On the other hand, small flocks relied on scavenging in the small-scale free-range and semi-intensive systems (Figure 4B).

Some of the feed ingredients used in formulating own-feed ration were produced within the region and sold at low prices including maize bran, maize grain, and sunflower seedcake, sold at 238, 259, and 349 Tanzania Shilling (**TZS**) per kg, respectively. Other feed ingredients imported from other regions/countries were expensive including the premixes, fishmeal, soybean meal, salt, bone meal, limestone, and cottonseed cake sold at TZS 3639, 1824, 1801, 1000, 733, 655, and 325 per kg, respectively. Soybean was produced within the region, but the local processing facilities rely on crushing which results in a poor quality feed with a large oil content which is not suitable for chicken. As the result, the soybean grain produced was exported to the neighbouring countries (sold at around TZS 800 per kg) for processing whereas soybean meal was imported at more than double the price of TZS 1,801 per kg. Some of the major feed ingredients used by farmers, such as maize and maize bran and sunflower seedcake, were mainly available on a seasonal basis leading to an increase in feed prices and costs of production particularly during the rainy season.

#### Breeding and Access to Day-Old Chicks

Farmers indicated that they experienced limited availability of day-old chicks, particularly for broilers, since they had to wait for a long time to receive them after placing their orders to the hatcheries through their agents in the region. This problem was mostly faced by commercial medium-large scale intensive farms as they work in cycles replacing all adult chickens at the same time. Despite the consumer demand for indigenous chickens, there were no hatcheries specialized in providing a large number of indigenous day-old-chicks.

#### Access to Market for Chicken and Chicken Products

**Figure 7:**
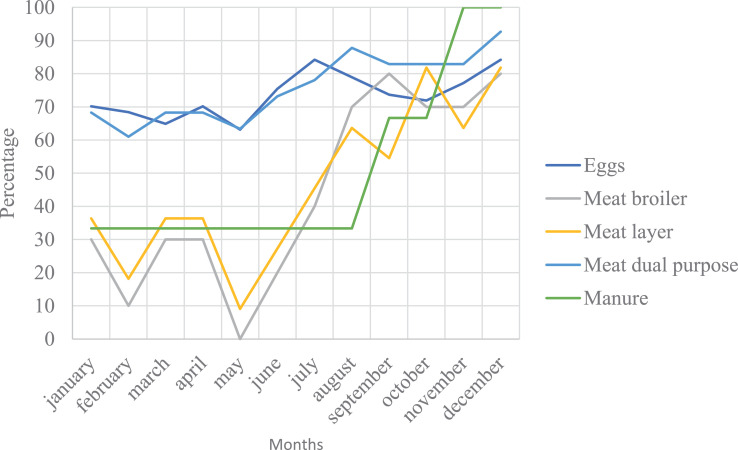
Percentage of the respondents that agreed that live chicken and their product markets were readily available in different months in the Iringa region.

The market for eggs and meat of the dual-purpose chicken was available throughout the year (Figure 7). However, there were seasonal fluctuations in selling the products from the exotic breeds (layers and broilers) from January until August. The peak market for all products was reported in December mainly related to the end of the year celebrations, and the worst market for broiler and layer's meat reported from January to July. Limited access to market for the small-scale free-range farmers (Figure 5), was partly associated with lack of farmers organizations to aggregate their produce. Chicken manure was also among the important outputs from the chicken farm, with larger market demand from mid-September to December, mainly applied in own-farms.

Based on the field visits and interviews, we found that there were no proper marketing infrastructures in the region, with no professional hygienic chicken slaughtering and storage facilities within the regional market. Chicken customers could buy a live chicken and slaughter it at home and/or have it been slaughtered at the marketplace in urban locations, where there was poor infrastructure with a high risk of contamination.

### Options for Improving Domestic Production of Meat and Eggs in the Identified Systems

**Figure 8:**
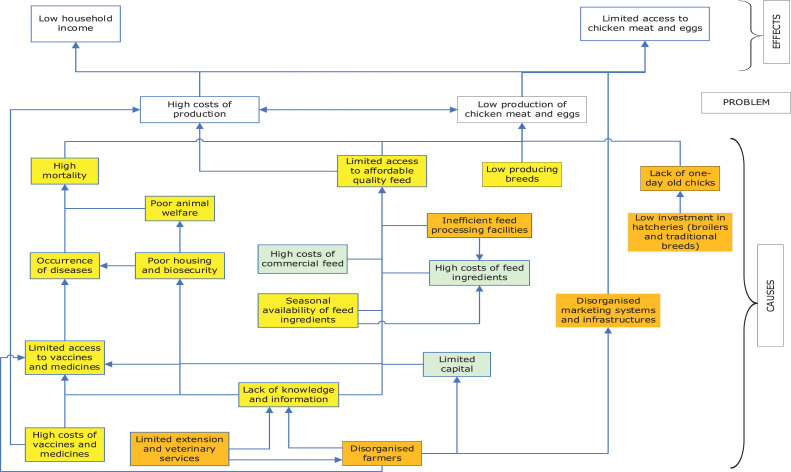
Constraints limiting chicken production in Iringa region categorized into financial, technical, and institutional constraints indicated by green, yellow, and orange boxes, respectively.

The relationships among, and consequences of the identified financial, technical, and institutional constraints were further explored by building a problem tree (Figure 8). This also allowed us to identify missing links. The final analysis was used to identify options to improve domestic production and productivity of chickens in the different farm types in the region. The identified constraints led to more general advice for the respective farm types. We found that the development of the chicken industry is constrained by high costs of production and low productivity of chickens. These two problems lead to limited access to chicken meat and eggs and low income for the farming household. The core causes of the identified problems were categorized into three clusters namely technical, institutional, and financial. The technical constraints limiting the development of the chicken industry link to both institutional and financial constraints. These include limited access to quality feeds associated with lack of capital, seasonal availability of feed ingredients, and limited knowledge to formulate feed rations using the locally available ingredients, particularly for the smallholder farmers. Other technical constraints included high mortality of chickens due to poor managerial practices, lack of knowledge, lack of farmer groups, limited extension services, and high costs of vaccines and medicines. The institutional constraints limiting access to quality feeds were partly related to the lack of suitable feed processing facilities for soybean grain in the region.

## DISCUSSION

### Chicken Farming Diversity

Numerous authors have characterized chicken farming under extensive production systems, but with limited differentiation and subdivision of farm types in urban and rural locations (Guni et al., 2013; Haoua et al., 2015; de Glanville et al., 2020). In the past 2 decades, studies in Tanzania have described chicken production systems but have not investigated or addressed the challenges that different farmers face that limit productivity and production. Most of the challenges reported were related to the existence of diseases and low production and productivity of chickens, limited access to feeds, markets, extension, and veterinary services (Buza and Mwamuhehe, 2000; Msami et al., 2006; Ngongolo and Chota, 2021). Our study confirmed an intensification gradient in chicken farming systems ranging from free-range/extensive production to semi-intensive and intensive systems in Tanzania. To better understand chicken farming diversity and associated constraints limiting production in urban and rural locations, we first made subdivisions within these farm types.

The subdivision of the farms in this study revealed differences in the degree of intensification from subsistence small-scale production to more market-oriented systems. The specific characteristics linked to intensification were based on the breed of chicken raised by farmers (indigenous, improved cross-bred and exotic breeds), rearing system (housing and feeding), flock size (instant number of birds), the purpose of keeping chicken and their productivity (meat and/or eggs). As a result, 5 farm types were identified in the region that is, small-scale free-range, small-scale semi-intensive, medium-scale semi-intensive, small-scale intensive, and medium-large scale intensive farms. The subdivision of different farm types in the current study allows us to differentiate which of the observed technical, institutional and financial constraints are most relevant for each farm type and further increases our ability to identify relevant intervention options for improvement in each of the farm types.

The intensification of chicken production systems in the Iringa region partly involved diversification through keeping multiple breeds of chicken in one or more rearing systems in the farming household. The households keeping exotic broilers and layers also raised the indigenous and/or improved crossbred both for meat and eggs. Diversified chicken systems have the potential for income generation through keeping the exotic and improved cross-bred with high productivity (Wolfgang, 2020) and meeting the household preferences for eggs and meat from the indigenous chickens (Naggujja et al., 2020). Diversification of chicken systems in the Iringa region is mostly found in the mid-large scale intensive system both in urban and rural locations where we found a large number of chickens and eggs both sold and retained for household consumption.

### The Intensification Gradient: From Subsistence to Medium-Large Scale Farming Systems

We found that the indigenous breed of chickens dominates the Iringa region, and that this breed was found even in the more intensive production systems. Nevertheless, the productivity of indigenous breeds was very low resulting in a small amount of the products (meat and eggs) retained for household consumption and/or sold to generate income. We found a transition from keeping indigenous chickens under the small-scale free-range system to both small-scale semi-intensive and intensive systems in rural locations. By contrast, in urban areas the transition is mainly toward the intensive system, partly related to limited space in urban areas as was also found in the capital city, Dodoma by (Ngongolo and Chota, 2021). Andrew et al. (2019) found that in Tanzania the indigenous chickens are mostly preferred by the highly risk-averse farmers due to their resistance to diseases and high survivability while raised with low costs. Furthermore, indigenous chicken meat and eggs are preferred by consumers compared with the meat from broilers and eggs from exotic layers in the country (Naggujja et al., 2020). Despite the strong consumer preference of indigenous chicken meat and eggs and the change from free-range to more intensive systems with indigenous breeds, it is challenging to meet the current demand through domestic production and supply (MLDF, 2015), because of the low productivity of the indigenous breeds.

In Iringa, we found that the degree of intensification is increasing with the number of improved crossbred and exotic chickens raised under the medium-large scale intensive systems and fed with homemade and/or commercial feeds in both urban and rural locations. We also found that intensive farms in urban locations raised quite a substantial number of indigenous chickens compared to the backyard and semi-intensive systems. This may be due to the assumed increased need of care for exotic breeds compared with indigenous breeds because of their greater susceptibility to diseases or because of their higher management costs (Andrew et al., 2019). The farms under this typology were commercially oriented whereby most of the produce was for sale. These are typical characteristics of commercial mid-large scale production systems in East Africa (Chaiban et al., 2020). Recent studies show that in Tanzania there is a transition to keeping improved dual-purpose breeds under semi-intensive and intensive systems mainly driven by high egg and meat production, high consumer appreciation of the products (Naggujja et al., 2020), high growth rate and high potential for income generation (Wolfgang, 2020).

Thus, in Iringa on one hand, the small-scale farms own small flocks under a low input-output system while relying on scavenging and/or partial feeding and producing their own chicks using natural hatching as for most developing countries (Pius et al., 2021). On the other hand, the medium-large scale farmers buy feed and feed ingredients as well as the 1-day-old chicks from the feed companies, hatcheries and/or selling agents (Wilson et al., 2021). Based on the subdivision of the farming systems, we found that while the intensification of small-scale farming systems is highly constrained by limited access to quality feed, the development of the medium-large scale farms is constrained by a limited supply of 1-day-old chicks. Despite higher degree of management, more intensive systems reported more disease problems (Figures 5 and 6) which may be due to the larger number and density of chickens, uniform in age and indoor housing in one farm compared with the extensive systems where more disease-resistant chicken scavenge freely outdoors.

### Linkages Between the Constraints in Different Production Systems and Practical Solutions

Based on the problem tree analysis we found that we need to consider multiple options at the same time when addressing the constraints limiting the growth of the chicken industry in Tanzania. Clustering the technical, institutional, and financial challenges explored possible solutions that might contribute to reducing the costs of production and ensuring a profitable chicken enterprise for each farm type and meeting the increasing demand for chicken meat and eggs (MLDF, 2015). The technical and institutional packages identified in the current study include investing in research on improving the genetic potential of the indigenous breeds of chickens, increasing the capacity of local hatcheries to reduce the shortage of 1-day-old chicks, particularly for the medium-scale semi-intensive and medium-large scale intensive farmers who keep the improved and exotic breeds of chickens.

Since 2016, Tanzania has banned the importation of chicken and poultry products but allowed the import of parent lines (both fertilized eggs and day-old chicks) for the local hatcheries to reduce the risk of spreading avian influenza into the country (Wilson, 2015; Naggujja et al., 2020). The import ban stimulated investment in strengthening the capacity of local hatcheries within the country and has contributed to reducing dependency on imports over the past 5 yr (Ringo and Lekule, 2020).

Moreover, it should be noted that the genetic strain of chicken is strictly linked with feed quality. High performance chickens need high quality feed, in particular high energy and protein concentrations. Thus, the use of the improved crossbred chickens could be more useful to smallholder farmers since they have lower nutrient requirements and are more disposed to use alternative feed sources compared to the exotic breeds. The recent introduction of the improved dual-purpose chickens (Sasso and Kuroiler breeds) has enormous potential due to their high genetic potential and adaptability in semi-scavenging systems (Sanka et al., 2020; Guni et al., 2021) that might be affordable for resource-poor farmers. The establishment of local hatcheries in the Iringa region is of great advantage to ensure a reliable supply of day-old chicks (Wilson et al., 2021). Nevertheless, there is a need to increase their capacity to reduce the seasonal lack of day-old chicks, including the exotic broilers serving the peak market from mid-September to December that we observed in the present study.

Feed costs account for up to 70% of the total costs of production in chicken farming (Mutayoba et al., 2011). Chickens are fed with highly nutritious and digestible energy and protein feeds including maize and fish meal (mainly sardines) which could be part of human food, implying food-feed competition (Wilson et al., 2021). Maize is produced by almost all smallholders in Tanzania and is readily available for chicken farmers. On the other hand, fish (sardines) and other protein sources were imported from other regions at high prices and their inclusion in chicken feeds was limited and contributed to high costs of production.

Improving access to affordable quality feed is an important pathway to reduce the costs of production and contribute to stimulating the commercialization of small-holder free-range production systems. Research is needed to explore options to reduce the seasonal feed shortages and find alternative feed sources that are not consumed by humans that is, alternative proteins including soybean meal, insects and insect larvae (Van Huis et al., 2020). Furthermore, research and training are crucial on proper feed formulation for diverse types/groups of chicken using the locally available feed ingredients. Soybean is produced in the region, but its use is hindered by limited processing facilities. And therefore, investing in efficient soybean processing facilities is of immense importance to improve the local availability of quality protein which is currently expensive in the region. Addressing the challenges related to feeds and feeding might contribute to ensuring a profitable chicken enterprise that would attract more youth and women to the poultry business (Hundie et al., 2019; Ngongolo and Chota, 2021). In turn, this would contribute to improving dietary diversity in the farming households through own-production and consumption and/or through purchase (Wilson et al., 2021). Packages with smaller quantities could also improve access to commercial feeds that were also expensive and packed in 50 kg bags.

The technical and institutional packages for reducing the occurrence of chicken diseases and mortality include strengthening the extension and veterinary services and training farmers on proper managerial practices and veterinary measures. For the intensive system, there is also a need to improve access to finance to enhance the construction of proper housing systems to accommodate large flock sizes while ensuring veterinary measures. We found that most vaccines were both expensive and packed in large doses that are less affordable for chicken farmers. An option would therefore be to invest in small doses of vaccines that might be affordable by small-holder and mid-scale farmers. Another option is to facilitate collective vaccination allowing large vaccine packages to be shared by more producers.

### Establishment of Farmer Groups and Contract Farming in Relation to Input-Output Markets

In the problem tree analysis we saw that lack of farmer organization was identified as a core cause of multiple constraints. We found that chicken farmers in our study were disaggregated and that most did not engage in farmer groups in both urban and rural locations contributing to the limited access to input and output markets. Since most vaccines are sold in large doses, and need cold chain transportation, organizing the smallholder farmers has the potential in reducing costs when a large number of chickens are vaccinated in one go (Campbell et al., 2019). Organizing farmers into groups can also strengthen group initiatives that is, access to soft loans from microfinance and access to improved inputs (i.e., resilient breeds of day-old-chicks and/or point of lay hens, housings, medicines), training and extension services on proper management practices, and access to information (Beesabathuni et al., 2018). Organizing farmers into groups may also shorten the marketing channels through direct sales of chickens and eggs to consumers and increase their equity and bargaining power in getting better prices for the products (Aklilu et al., 2007).

Many business models exist, related to the development of smallholder chicken farming systems in low- and mid-income countries (**LMICs**) with an emphasis on the aggregation of smallholder farmers to enhance trading partnerships and engage them with diverse actors that is, the NGOs, private business companies, microfinance institutions. The models also emphasize the training of farmers on proper management, feed processing, group marketing and large-scale rearing of chickens to ensure viable and profitable business. The viable business models implemented in LMICs include micro franchising with small capital investment for smallholder backyard farmers with small margins; microfinancing, and cooperative farming both targeting the commercialization of backyard farmers to raise more chickens and aggregating farmers into small groups (Beesabathuni et al., 2018).

Other business models proposed for the growth of smallholder farmers in LMICs include the outgrower and enterprise development. For the enterprise development model, the input supplier is responsible for organizing smallholder farmers into groups and coordinating the enterprise development. The support services provided by the input suppliers include the training, input packages on credit and organizing markets for the produce. In this model, farmers are encouraged to sell eggs to the local community and hence contribute to improving dietary diversity. Farmers are also aggregating the excess produce and transporting them to urban markets using the trucks used in delivering feeds in rural areas. In this model participating chicken farms may be of diverse types and may decide to select amongst the services provided and the pace at which they develop their business. On the other hand, the outgrower model has the same capital investments for the farmer and for the company. This model involves a formal contract between a commercial entity and an independent farmer or farmer group managing ≥5,000 chickens. The commercial entity supplies day-old-chicks, extension and veterinary services, input packages including feed, regular farm visits and monitoring of the farm. They can also select the local parties to whom they sell their products (Beesabathuni et al., 2018). The outgrower model was partly implemented in the southern highlands of Tanzania (Mugittu, 2016). Unfortunately, little is known yet about the progress and output of the project.

## CONCLUSIONS

Our study revealed a large diversity of chicken production systems in Tanzania, beyond what had been described previously. We found that the indigenous breed of chickens was dominant in the region and was found even in the more intensive production systems. We also found that about 30% of farms had multiple chicken production systems alongside each other. The intensification gradient in chicken production from subsistence small-scale production to more market-oriented semi-intensive and intensive systems was confirmed. Intensification involves the transition from keeping indigenous chickens to the improved dual-purpose chickens driven by high egg and meat production, high consumer appreciation of the products, high growth rate and a high potential for income generation. The subdivision in production systems and the problem tree analysis revealed different constraints limiting chicken production depending on the types which open the door to propose relevant packages for improving the production of meat and eggs for the household and other consumers. Despite a greater management input, more intensive systems reported more disease problems implying that there should be training on improved housing and veterinary measures, particularly for the farmers intensifying to the medium to large scale production systems. Apart from the existence of chicken diseases in all the systems, the development of the medium-large scale systems is highly constrained by a limited supply of one-day-old chicks and theft, while the intensification of small-scale systems is constrained by limited access to quality feeds, vaccines and medicines, capital, and reliable output market, partly associated with lack of farmer organization. There is therefore a need for institutional support to organize producers into groups and/or cooperatives with emphasis on training them on proper management, low-cost feed processing, group marketing, and large-scale rearing of chickens. The enterprise development might be the most promising business model for the growth of smallholder chicken farming since farmers have more freedom in farm management and decision making while they will be linked with the input suppliers and output markets to ensure a viable and profitable business.

## Appendix Supplementary materials

Image, application 1

Image, application 2
