# Supplementary material for: The diversity of smallholder chicken farming in the Southern Highlands of Tanzania reveals a range of underlying production constraints
Source: Poult Sci. 2022 Jul 26;101(10):102062. doi: 10.1016/j.psj.2022.102062 (PMC9441339; doi:10.1016/j.psj.2022.102062)
Supplement: Supplementary file 1 [file mmc1.docx]

**SUPPLEMENTARY FILES**

Table S1. Number of chickens raised, chicken products consumed and sold in urban and rural areas of the studied study area

|  | | **Farm location** | |
| --- | --- | --- | --- |
|  |  | **Urban (n=48)** | **Rural (n=73)** |
| **Number of chickens per household (mean and range)** | Layers^1^ (n=21) | 482.80 (9-5700) | 278.00 (30-600) |
|  | Broilers^1^ (n=14) | 378.00 (15-994) | 220.83 (47-400) |
|  | Dual purpose^1^ (n=110) | 106.27 (7-500) | 51.10 (2-300) |
| **Products for sale and household consumption** | Egg production/week^1*^ | 550.45 (4-11970)^a^ | 74.91 (0-950)^b^ |
|  | Eggs sold per week^1*^ | 496.11 (0-11940) ^a^ | 58.29 (0-900) ^b^ |
|  | Eggs retained for home consumption/week^1^ | 11.89 (0-30) ^a^ | 6.76 (0-30) ^b^ |
|  | Chicken sold/month^1^ | 17.97 (0-167) ^a^ | 8.29 (0-61) ^b^ |
|  | Chicken retained for home consumption/month^1^ | 1.80 (0-18) | 1.22 (0-5) |

^1^mean and minimum and maximum number

Different superscripts (a,b) indicates a significant difference between urban and rural at p-value ≤ 0.05 level
