# Supplementary material for: The diversity of smallholder chicken farming in the Southern Highlands of Tanzania reveals a range of underlying production constraints
Source: Poult Sci. 2022 Jul 26;101(10):102062. doi: 10.1016/j.psj.2022.102062 (PMC9441339; doi:10.1016/j.psj.2022.102062)
Supplement: Supplementary file 2 [file mmc2.pdf]

Table S2. Description of the households with multiple breeds/types of chickens in different systems in the Iringa region

	Breed X rearing system								
	Indigenous free-range	Indigenous semi-intensive	Indigenous intensive	improved crossbred_free-range	improved crossbred_semi-intensive	improved crossbred_intensive	exotic_free-range	exotic_semi-intensive	exotic_intensive
1		X						X	
2			X						X
3		X			X	X			
4			X			X			X
5									X
6						X			
7									X
8			X						
9	X					X			
10			X			X			
11			X						
12		X			X	X			
13	X								
14	X								
15	X		X						
16			X						
17		X							
18		X			X	X			
19									X
20						X			X
21						X			X
22			X			X			
23			X			X			X
24			X			X			X
25			X			X			
26	X					X			
27	X					X			
28	X					X			
29	X					X			
30						X			X
31		X							
32			X			X			
33			X			X			
34			X						X
35	X								
36		X							
37			X						
38			X						
39	X								
40			X						
41			X						

42			X			X			
43		X							
44	X					X			
45			X						
46	X					X			
47									X
48			X						
49	X		X			X			
50	X	X							
51			X						X
52	X					X			
53	X	X							
54	X					X			
55	X								
56	X								
57	X					X			
58	X								
59									
60									X
61			X						
62									
63									X
64	X								
65			X						
66	X								
67	X								
68			X						
69		X							
70									
71									
72									
73			X						
74									X
75									X
76		X							
77									
78		X							
79	X	X							
80		X							
81		X							
82	X					X			
83		X							
84		X			X				
85		X							
86		X							
87	X								
88			X			X			
89	X								
90	X								
91	X								

92	X								
93									
94			X						
95	X								
96									
97			X						
98									
99			X						
100	X								
101	X	X							
102		X							
103		X							
104			X						
105	X					X			
106		X							
107		X							
108		X							
109		X							
110		X							
111	X								
112	X		X						
113									
114	X								
115		X							
116									
117			X						
118									
119	X								
120	X								
121		X							

Type of chicken X rearing system								
egg-type free-range	egg-type semi-intensive	egg-type intensive	dual-purpose_free-range	dual-purpose_semi-intensive	dual-purpose_intensive	meat-type free-range	meat-type semi-intensive	meat-type_intensive
				X			X	
		X			X			X
	X			X			X	
		X			X			X
		X			X			X
								X
		X			X			X
					X			X
			X			X		
					X			
				X				
			X					
			X					
			X					
			X					
				X				
				X				
		X			X			
					X			
					X			
		X			X			
		X			X			
		X			X			
					X			
			X					
			X					
			X					
		X			X			
					X			
					X			
		X			X			
			X					
				X				
					X			
					X			
					X			

					X			
			X					
					X			
			X					
		X			X			
					X			
			X		X			
			X	X				
					X			
			X					
			X	X				
			X					
			X					
			X					
			X					
			X					
					X			
		X			X			
					X			
					X			
		X						
			X					
					X			
			X					
			X					
					X			
				X				
					X			
					X			
					X			
		X			X			
		X			X			
		X			X			
				X				
					X			
				X				
			X	X				
				X				
				X				
			X					
				X				
				X				
				X				
				X				
			X					
					X			
			X					
			X					
			X					

			X					
		X			X			
					X			
			X					
		X			X			X
					X			X
		X			X			X
					X			X
			X			X		
			X	X				
				X				
				X				
					X			
			X					
				X				
				X				
				X				
				X				
				X				
			X					
			X		X			
		X			X			
X			X					
					X			
					X			
		X			X			
			X					
			X					
				X				
